# The Association between the Gut Microbiome and Development and Progression of Cancer Treatment Adverse Effects

**DOI:** 10.3390/cancers15174301

**Published:** 2023-08-28

**Authors:** Amanda S. Maddern, Janet K. Coller, Joanne M. Bowen, Rachel J. Gibson

**Affiliations:** 1 School of Allied Health Science and Practice, The University of Adelaide, Adelaide, SA 5005, Australia; amanda.maddern@adelaide.edu.au; 2 School of Biomedicine, The University of Adelaide, Adelaide, SA 5005, Australia; janet.coller@adelaide.edu.au (J.K.C.); joanne.bowen@adelaide.edu.au (J.M.B.)

**Keywords:** gut microbiome, chemotherapy, radiotherapy, chemoradiotherapy, cancer treatment, adverse effects

## Abstract

**Simple Summary:**

Treatment of cancer can cause a multitude of unwanted effects including nausea, vomiting, diarrhoea, fatigue, cognition changes and weight loss. We also know that the microbiome of the gut can be altered by cancer treatments. In this review we investigated the existing evidence from human studies that supported a link between changes in the gut microbiome and the occurrence of the unwanted effect. We found that whilst there is some evidence linking gut microbiome and nausea, vomiting and diarrhoea, more research with people undergoing cancer treatment is required to expand our understanding and to investigate if modulation of the gut microbiome can be an effective treatment for unwanted effects.

**Abstract:**

Adverse effects are a common consequence of cytotoxic cancer treatments. Over the last two decades there have been significant advances in exploring the relationship between the gut microbiome and these adverse effects. Changes in the gut microbiome were shown in multiple clinical studies to be associated with the development of acute gastrointestinal adverse effects, including diarrhoea and mucositis. However, more recent studies showed that changes in the gut microbiome may also be associated with the long-term development of psychoneurological changes, cancer cachexia, and fatigue. Therefore, the aim of this review was to examine the literature to identify potential contributions and associations of the gut microbiome with the wide range of adverse effects from cytotoxic cancer treatments.

## 1. Introduction

The gut microbiome is a highly complex ecosystem comprised of both aerobic and anaerobic species [1,2]. It plays key roles and functions vital to healthy states, including but not limited to, epithelial protection, metabolism of different enzymes, processing of nutrients, regulation of gastrointestinal (GI) angiogenesis, and interactions with the immune system [3]. In recent years, the gut microbiome was the focus of much attention for its potential role in many human diseases, including but not limited to, obesity [4], Alzheimer’s disease [5], and non-alcoholic steatohepatitis [6]. However, a particular area of focus is on the role the gut microbiome may play in many aspects of supportive care during cancer treatment.

Along with the well-established variations in the gut microbiome between individuals with different ethnicities, age, sex, and diet [7], the gut microbiome composition has been shown to influence cancer prognosis [8], treatment efficacy [9], and outcomes post-treatment [10]. Specifically, research has consistently shown that cytotoxic cancer treatments alter gut microbiome composition and reduce diversity [2], which can influence the physiological response(s) to cancer treatments, including chemotherapy (CT), radiotherapy (RT), and immunotherapy, and their resulting sequalae [11,12,13,14,15,16]. Common adverse effects of these treatments include GI [12,13] and oral mucositis [11], fatigue [17,18], psychoneurological changes including anxiety, fear of cancer recurrence and altered cognition [19,20], and cancer cachexia [21,22]. Whilst there is substantial evidence supporting the role of the gut microbiome in the development and progression of GI and oral mucositis [11,16,23,24,25], there is less evidence investigating the potential impact that the gut microbiome has on the development of psychoneurological changes, cancer cachexia, and fatigue. Consequently, this review aimed to interrogate the existing literature for evidence implicating the association between the gut microbiome and development and progression of cancer treatment adverse effects. Briefly, a semi-structured literature search for relevant papers was conducted using PubMed, Embase, Emcare, and Cochrane databases. For the purposes of this review, studies were limited to clinical research studies only. Whilst preclinical studies are outside the scope of this review, for a comprehensive update, please see Bowen and colleagues [26]. The list of key search terms is provided in the Supplementary Table S1. Papers were reviewed by all authors and key aspects regarding participants, methodology, and outcomes from each study were synthesised as per the aim of the review. Whilst we are cognisant of other roles of the gut microbiome in cancer prognosis and treatment efficacy as mentioned above, this was considered outside the scope of this review.

### 1.1. The Gut Microbiome

**Figure 1:**
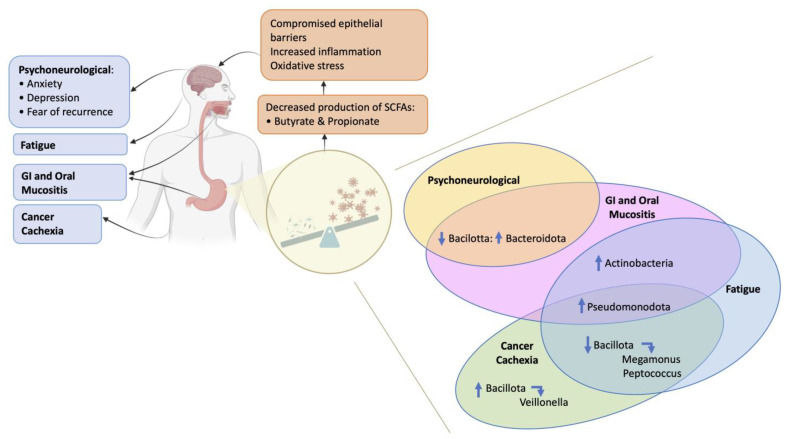
The potential impact of gut microbiome dysbiosis during and after cancer treatment on changes in physiological mediators and processes and adverse effects; and the relative abundance changes in specific gut microbiome taxa with each specific adverse effect. Abbreviations: GI—gastrointestinal; SCFAs—short chain fatty acids. Up arrows indicate an increase and down arrows a decrease in abundance of taxa; side arrows indicate consequent changes in taxa within that family.

The gut microbiome is an overarching term that encompasses all regions of the gut. However, as the majority of clinical trials investigate faecal samples, this represents mostly the composition of the colon and so, findings are often generalised to that region [27]. The gut microbiome is a highly complex ecosystem collection of bacteria, viruses, and microorganisms, all of which contribute to, and ultimately impact, many physiological processes [13,14,15,28]. Specifically, this includes protection against pathogens, the metabolism of nutrients, maintenance of the integrity of the intestinal mucosal barrier, and immune regulation and response [13,15]. In healthy states, the gut microbiome is highly diverse, consisting of 100 trillion bacterial and archaeal cells and involves more than 1000 different species [28]. Specifically, there are many different types of bacteria within the gut microbiome; the most abundant phyla are *Bacteroidetes, Firmicutes* (together accounting for 90% of the microbiome in healthy states), *Actinobacteria,* and *Proteobacteria*. It is broadly considered that when the balance of bacteria within the gut microbiome is stable, homeostasis is maintained between the gut and the microbiome [13,15]. However, when the delicate balance is disrupted, dysbiosis occurs [13,15,29] and may subsequently lead to a range of negative health effects, including but not limited to, increased inflammation [29] and potentially tumourigenesis [15]. Further, the composition of the gut microbiome may impact both development and progression of short- and long-term sequalae related to cancer treatments [16,18,22,30,31], ultimately impacting quality of life during treatment and survivorship (Figure 1).

Whilst a detailed discussion of the mechanisms underlying the gut microbiome modulation of cancer treatment adverse effects, including CT, RT, and immunotherapy, is outside the scope of this review, one potential modulator is likely to be short chain fatty acids (SCFAs); for a detailed review, please see Al-Qadami et al., 2022 [14]. In brief, SCFAs are metabolites produced by the gut microbiome with acetate, butyrate, and propionate being the most common [14]. These SCFAs have a range of functions within the gut, including immune modulation and stabilising epithelial barriers [14]. Acetate is produced in high levels as a direct consequence of the many different gut bacteria able to produce it [14]. In contrast, butyrate and propionate production is limited to certain bacterial types, including the *Firmicutes* and *Bacteroidetes* phyla [14,32], and therefore is not found in high levels. Gut dysbiosis may lead to reduced production of SCFAs, compromising epithelial barriers and increasing inflammation and oxidative stress [14]. These changes in the production of SCFAs may therefore significantly impact the adverse effects [14]. Indeed, evidence now suggests SCFAs also have many protective, anti-inflammatory, and anti-oxidant properties [33], and as such may be new targets to explore in the management of these adverse effects [14]. In addition, there is a plethora of research suggesting that the gut microbiome has an important role in CT, RT, and immunotherapy efficacy [15,34], a detailed discussion of which is outside the scope of this paper; for an excellent review please refer to Liu and Shah 2022 [35]. However, in brief, a landmark paper by Alexander and colleagues [36] proposed the TIMER (**T**ranslocation, **I**mmunomodulation, **M**etabolism, **E**nzymatic degradation, and **R**educed diversity and ecological variation) mechanistic framework as a potential explanation for how the gut microbiome may specifically influence cancer treatment efficacy. Using this framework, they suggested the gut microbiome is a critical target to be exploited in a type of personalised medicine approach to improving cancer treatment efficacy as well as minimising adverse effects. More recently, this work was extended with an excellent review by Huang and colleagues [37]. Briefly, they reviewed the literature and identified the human microbiota as a possible biomarker for the prediction of treatment efficacy. Further, they proposed a number of strategies, including faecal microbiota transplants and probiotics, which may be effective in improving adverse effects.

### 1.2. Changes in the Gut Microbiome as a Consequence of Cancer Treatment

As described above, cancer treatments are known to significantly alter the gut microbiome, resulting in changes in abundance of key bacteria; for example, an increase in harmful bacteria including *Proteobacteria* and *Fusobacteria,* coupled with a decrease in beneficial bacteria including *Faecalibacterium* and *Bifidobacterium* [15]. Our knowledge of these specific changes has been possible with the advances in sequencing technologies including 16S rRNA sequencing and deep metagenomic sequencing now being commonly utilised in clinical research. One of the early studies to do this was in a 2016 study by Rajagopala and colleagues, who examined the gut microbiome of 51 matched paediatric and adolescent participants with acute lymphoblastic leukemia and their healthy sibling via faecal samples collected before, and at varying timepoints during, CT [38]. The 16S rRNA gene sequencing demonstrated that although the microbiome profiles were similar between groups with *Bacteroides, Prevotella* and *Faecalibacterium* being heavily represented, there was a significantly lower diversity within the acute lymphoblastic leukemia population. Specifically, the microbiome profiles were able to clearly delineate the groups and the study concluded these changes were most likely due to the direct influence of chemotherapeutics [38]. More recently, further support for changes in the gut microbiome were illustrated by El Alam and colleagues, who analysed gut microbiome changes in 58 women with newly diagnosed gynaecological tumours [39]. Rectal swabs were collected prior to commencing, and then at various timepoints during, and at 12 weeks following pelvic chemoradiotherapy (CRT) were analysed using 16S rRNA genomic sequencing. Results indicated a significant decrease in gut microbiome diversity during treatment, which only returned to baseline in just over half of the participants by 12 weeks post-CRT, and both the structure and composition of the gut microbiome remained altered from baseline [39]. These are just two studies of a plethora to provide clear evidence the gut microbiome changes in response to cytotoxic cancer treatments. It is these changes that are likely to be key in modulating associations between the gut microbiome and adverse effects following cancer treatment that will now be discussed.

## 2. The Gut Microbiome in Supportive Care during and after Cancer Treatment

There is a large evidence base supporting the influence of the gut microbiome over the acute adverse effects of cancer treatments, including the development of GI mucositis [13,23,24]. However, there is only emerging evidence regarding the potential influence that the gut microbiome might have over long-term adverse effects including psychoneurological changes [20], including anxiety and fear of recurrence [19], cancer cachexia [21,22], and fatigue [17,18]. Adverse GI effects are extremely common after cancer treatments [13,14,15], leading to a wide range of symptoms, including ulceration, diarrhoea, pain, and malnutrition in approximately 50% of all patients with cancer [40,41]. These symptoms can be so severe that they result in treatment dose reductions and delays, which may ultimately lead to reduced treatment efficacy and impact survival outcomes [41]. Over recent years, there have been a number of studies that implicated the gut microbiome in the development and subsequent progression of GI adverse effects following cytotoxic cancer treatment. We identified 10 papers that investigated the potential role of the gut microbiome in the development of oral and GI mucositis arising from cancer therapy; seven studies specifically investigated GI mucositis [23,24,25,30,42,43,44], one study investigated both GI mucositis and fatigue [17], and the remaining two investigated oral mucositis [11,16]. Table 1 summarises the key aspects regarding participants, methodologies, and outcomes for each of these papers, which are discussed in turn below.

### 2.1. Gastrointestinal (GI) Mucositis Following Chemotherapy (CT)

Three studies specifically investigated the role of the gut microbiome on the development of GI mucositis in participants undergoing CT [23,30,44] (Table 1). One of the first clinical studies to investigate the association between CT-induced diarrhoea and changes in the gut microbiome was conducted by Stringer and colleagues in 2013 [23]. This small study recruited 26 participants with a variety of solid tumours. It was the first to observe changes in faecal microflora in participants with diarrhoea as a consequence of CT; however, significance was not obtained due to the small size. Importantly, these changes coincided with a significant elevation in faecal calprotectin and serum MMP-3 and -9 levels. These findings were the first to suggest that CT-induced diarrhoea was associated with changes to the gut microflora and hypothesised that this may result in diminished gut function, leading to the onset of this symptom. In 2015, Montassier and colleagues examined the gut microbiome in 28 patients prior to, and following, CT for non-Hodgkin’s lymphoma [30]. All patients in this study reported GI mucositis following CT treatment. Briefly, faecal samples were analysed through 16S rRNA sequencing, and similar to Stringer and colleagues [23], changes in the gut microbiome were observed. Specifically, there were decreases in the abundance of the Firmicutes and Actinobacteria phyla coupled with increases in abundance of the Proteobacteria phylum following CT, and the authors suggested these changes in the microbiome may subsequently be implicated in the development of GI mucositis. More recently, Aarnoutse and colleagues investigated changes in the gut microbiome at varying time points following CT in 44 participants with breast cancer [44]. Faecal samples were collected and analysed with 16S rRNA sequencing with a decrease in the bacterial richness, diversity, and composition of the gut microbiome in all participants, including reduced abundance of *Proteobacteria* and *Lactobacillus* identified. Further, participants who developed any diarrhoea had lower species richness compared to those participants who did not develop diarrhoea. Taken together, these studies provide strong evidence for the role of the microbiome in the subsequent development of GI mucositis following CT.

### 2.2. Gastrointestinal (GI) Mucositis Following Radiotherapy (RT) or Chemoradiotherapy (CRT)

Four studies specifically investigated the role of the gut microbiome, via 16S rRNA sequencing and/or DNA fingerprinting, in the development of GI mucositis in participants undergoing radiotherapy (RT) or chemoradiotherapy (CRT) (Table 1). The first of these studies was in 2008, when Manichanh and colleagues recruited 10 participants who underwent RT for mixed abdominal tumours [42]. Briefly, faecal samples were collected before, during, and at the end treatment and analysed for microbiome changes. The six participants who subsequently developed diarrhoea had decreased bacterial richness, diversity, and composition compared to the four participants who did not develop diarrhoea. In 2015, Wang and colleagues collected faecal and blood samples from 11 participants undergoing pelvic RT pre- and post-treatment [17]. Similar to Manichanh and colleagues, they observed differences in microbial diversity and composition at both time points between participants who experienced diarrhoea compared to those who did not. In particular, there was a lower alpha diversity and higher *Firmicutes:Bacteriodetes* ratios along with a lower abundance of Clostridium XI and XVIII, Faecalibacterium, Oscillibacter, *Parabacteroides,* and *Prevotella*. Consequently, the authors concluded a close association was apparent between gut microbial dysbiosis and diarrhoea following pelvic RT. Four years later, a study investigated alterations in the gut microbiome in faecal samples prior to and following pelvic RT in 18 females with cervical cancer [24]. Microbial dysbiosis was associated with radiation enteritis, with reduced alpha diversity compared to those without radiation enteritis. However, in contrast, there was increased beta diversity with increased *Proteobacteria* and *Gammaproteobacteria* and decreased *Bacteroidetes* and *Firmicutes.* Also published in 2019, a much larger study was conducted by Reis Ferreira and colleagues that recruited 134 men undergoing RT for prostate cancer and assessed for early and late RT sequelae using faecal samples [25]. In addition, both clinician and patient-reported outcomes were collected. Similar to previous studies, the gut microbiome decreased in diversity in those participants with early RT-induced patient reported adverse effects; however, no other associations were observed. Finally, in 2020, Shi and colleagues investigated the association of the gut microbiome with GI adverse effects in 22 participants with rectal cancer undergoing CRT, with faecal samples being collected prior to and just after CRT [43]. Unlike the previous studies, no differences were seen in the richness or diversity between those with severe diarrhoea and those with no/mild diarrhoea; however, differences were seen in several taxa, including a decreased abundance of *Butyricicoccus* and *Hungatella*. The authors concluded these bacterial species may be subsequently used to predict GI adverse effects in this clinical population [43]. Taken together, these studies demonstrate significant changes in microbial richness, diversity, and composition in participants with GI adverse effects as a result of RT or CRT therapy, highlighting the potential clinical relevance of the gut microbiome as a possible predictive biomarker.

### 2.3. Oral Mucositis Following Cancer Treatment

Oral mucositis (OM) is an extremely common adverse effect of cancer treatments [11,16,45]. The oral cavity is more easily accessible, and thus the reporting and investigation of this adverse effect is more comprehensive than GI mucositis. There was a systematic review (*n* = 13 prospective clinical trials) and two clinical studies that investigated the potential role of the microbiome in the subsequent development of OM (Table 1). Importantly, these studies examined both the oral microbiome and gut microbiome. In 2007, Napeñas and colleagues undertook the systematic review investigating whether the microbiome of the oral cavity was associated with the development of OM following CT [46]. Thirteen studies were identified for inclusion in the review; however, they reported substantial variations within these studies with regards to differences in participant populations, sample collections, and sample sites. Ultimately, they were unable to draw a consensus between changes in the oral microbiome and subsequent OM development [46]. Six years later in 2013, Ye and colleagues investigated whether oral bacterial diversity and dynamics in 37 paediatric participants receiving CT were associated with OM [11]. Briefly, they reported participants who subsequently developed OM had higher oral microbial diversity and heterogeneity prior to CT [11]. Further, participants who developed OM had a greater change in microbial diversity during treatment, including increased abundances of *Fusobacteria* and *Spirochaetes* compared to those who did not develop OM [11]. Most recently, Al-Qadami and colleagues identified that the gut microbiome is associated with OM in participants with head and neck cancer [16]. They observed differences in gut microbial composition between participants who went on to develop severe OM (grade 3–4) following CT or CRT compared to mild OM (grade 1–2), namely higher abundances of *Eubacterium, Victivallis,* and *Ruminococcus* [16]. Further, there was a positive correlation between the relative abundance of *Victivallis* and the severity of OM [16]. Taken together, although it remains unclear exactly what taxa might contribute to the development of OM, these studies demonstrate that CT, RT, or CRT result in the dysbiosis of the oral and gut microbiome, ultimately leading to adverse effects.

## 3. Psychoneurological Disorders Following Cancer Treatments

The gut–brain axis is a bidirectional pathway that can be significantly altered by cancer treatments and potentially the composition of the gut microbiome [47,48]. Although the exact mechanisms of how the gut microbiome may influence neurological conditions remains unclear, proposed pathways include the ability of the microbiome to impact the central nervous system (CNS) through the vagus nerve, modulation of immune responses, and synthesis of metabolites including SCFAs [49], that are able to stimulate neurotransmitter release to further impact neural behaviour and signalling [50]. There is extensive literature suggesting that an alteration to the diversity and composition of the gut microbiome is associated with fatigue, cognitive problems, and mood changes [20,51]. Therefore, it is not unreasonable to suggest that dysbiosis of the gut microbiome leads to altered SCFA production and potentially altered neurological conditions [20,48]. Cancer treatments are also known to lead to changes in cognition and mood due to increased CNS inflammation and increased production of cytokines and chemokines [20]. Thus, the composition of the gut microbiome might provide a vital biomarker for early intervention for psychoneurological disorder treatment adverse effects. We identified six studies investigating the composition and impact of the gut microbiome on the psychoneurological effects of cancer treatments, including fear of cancer recurrence [19,20], depression and anxiety [20,31,52,53], and cognition [20,52]. Table 2 summarises the key aspects regarding participants, methodologies, and outcomes for each of these papers, which are discussed in turn below.

Okubo and colleagues provided the first evidence of an association between the gut microbiome in faecal samples and fear of cancer recurrence in 126 breast cancer survivors [19]. Briefly, in those participants that received CT, changes at the phylum and genus level, and diversity, were associated with fear of cancer recurrence. For example, at the phylum and genus levels, a higher abundance of *Bacteroides* was associated with a higher fear, whilst a higher abundance of *Firmicutes* at the phylum level, or *Lachnospiraceae.g*, *Ruminococcus* at the genus level, or higher alpha diversity was associated with a lower fear of cancer recurrence. The authors concluded this was a direct result of CT as no such changes were associated in participants that did not undergo CT.

Four studies investigated gut microbial composition on depression and anxiety in cancer survivors [20,31,52,53], with a variety of results reported. The first of these was conducted in 2017 by Paulsen and colleagues, who investigated the impact of gut microbiome changes in 12 breast cancer survivors following CT and RT on depression and anxiety as part of a larger study on fatigue [31]. Whilst they found an association between beta diversity and anxiety, they did not observe any other associations between microbiome composition and psychoneurological adverse effects. Five years later, Deleemans and colleagues investigated CT effects on the association between gut microbiome and various psychosocial adverse effects, including depression and anxiety, in survivors of mixed cancers who predominantly received CT, and compared them to healthy controls [52]. Survivors experienced higher anxiety, depression, PTSD, pain and social isolation, and lower cognitive function. With regard to microbiome differences, in survivors, alpha diversity was negatively correlated with depression and positively correlated with cognition [52]. Bilenduke and colleagues followed this study by also investigating differences in various adverse effects, including depression, cognition and stress perception, and the gut microbiome in female breast cancer survivors post-CT compared to healthy controls [20]. Similar to the previous study, survivors had higher depression and lower cognitive function and decreased relative abundance of the *Verrucomicrobia* phylum and the genus *Akkermansia*. Other changes were reported; however, the significance of these was not described, and overall, the authors concluded that there was evidence of an association between the gut microbiome and depression and cognition [20]. Most recently, these outcomes were supported by Smith and colleagues, who also found negative correlations between psychoneurological adverse effects and taxa abundance in female breast cancer survivors [53]. This included increased relative abundances of *Ruminococcus* and *Dorea* correlating with poorer mental health, physical activity, and vitality. Taken together, these studies began providing valuable insights into the potential role of the gut microbiome in long-term psychoneurological adverse effects following cancer treatments. However, in comparison to the large volume of evidence implicating the link between the gut and oral microbiome and GI adverse effects, it is clear the evidence in this space is in its infancy and further clinical studies are now warranted.

## 4. Cancer Cachexia Following Cancer Treatments

Cancer cachexia is a further adverse effect of cancer treatment that can lead to poorer response and long-term outcomes, and significantly reduced quality of life [22]. Although the underlying pathology is complex, cancer cachexia is primarily characterised by significant weight loss, specifically due to loss of skeletal muscle [22]. Current research suggests the development of cancer cachexia is potentially modulated by inflammatory cytokines including tumour necrosis factor alpha (TNF-α), interleukin (IL)-1, and IL-6, which may ultimately influence appetite regulation through reduced food intake and suppression of appetite [22]. For a thorough description of the factors that could potentially induce cancer cachexia, please refer to the recent review by Cao and colleagues [54]. Emerging research now suggests the gut microbiome may play a potential role in both the prevention and/or management of cancer cachexia due to the significant role it plays in physiological processes such as metabolism [13,22]. We identified three recent studies that investigated the association between the gut microbiome and cancer cachexia. Table 3 summarises the key aspects regarding participants, methodologies, and outcomes for each of these papers, which are discussed in turn below.

Ni and colleagues were the first to investigate these associations in baseline faecal samples from 31 cachectic and non-cachectic lung cancer patients [55]. Whilst they did not observe any differences in alpha diversity or the *Firmicutes/bacteroidetes* ratio, they did observe 44 species differences, including lower abundance of *Prevotella copri*. This study was shortly followed by Ubachs and colleagues’ much larger study that examined the microbiome of baseline faecal samples of 33 cachectic and 74 non-cachectic participants with a range of cancers and 76 healthy controls [21]. Similar to the previous study, no difference in the alpha diversity was observed; however, there were differences in various phyla and genus in the cachectic group compared to the other groups, including increases in Proteobacteria, *Veillonella,* and an unknown Enterobacteriaceae family genus member, decreases in *Megamonas* and *Peptococcus*, and altered co-occurrence of these taxa. Additionally, they reported lower levels of the SCFA acetate in the cachectic compared to the non-cachectic group that was negatively correlated with the abundance of *Peptococcus* and *Enterobacteriaceae* (unknown), linking back to the importance of SCFAs as a potential mediator. Most recently, Hakozaki and colleagues conducted a study of baseline faecal samples in 57 cachectic and 56 non-cachectic participants with lung cancer who were receiving immunotherapy with and without CT [22]. Again, they did not observe any difference in alpha diversity, but did report differences in beta diversity and various taxa of both higher (including the commensal bacteria *Escherichia-Shigella* and *Hungatella*) and lower (including *Anaerostipes* and *Blautia*) abundance between the groups. Taken together, the common findings of these studies between cachectic and non-cachectic participants were no differences in alpha diversity, but various other differences in microbial composition, with no clear and consistent changes. Consequently, the infancy of these investigations, similar to those in the psychoneurological adverse effects, necessitate further studies to replicate these recent findings and elucidate more fully the potential influence that the gut microbiome might have over the development and progression of cancer cachexia.

## 5. Fatigue Following Cancer Treatments

Fatigue is an incapacitating adverse effect of cancer treatment [18]. Though the underlying mechanisms are unclear, it is possible both CT- and RT-related fatigue may be influenced by the gut–brain axis [17,18] in a similar manner to other psychoneurological adverse effects [18]. Dysbiosis in the gut microbiome may also lead to changes in immune responses and the synthesis of metabolites, including SCFAs; which, as described above, both influence neuronal behaviours and signalling, including fatigue pathways [18,49]. We identified six studies since 2017 that investigated the association between the gut microbiome and fatigue following CT, RT, or CRT. Table 4 summarises the key aspects regarding participants, methodologies, and outcomes for each of these papers, which are discussed in turn below.

Of these studies that examined the influence of the gut microbiome on cancer treatment-related fatigue, all reported some differences in microbial composition between the participants with and without fatigue [18,31,52,56,57,58]. The first of these was a study in 12 breast cancer survivors that reported fatigue being associated with higher alpha and beta diversity [31]. This was followed by three studies in 2021, starting with Hajjar and colleagues in 88 participants with mixed cancers [56], who in baseline faecal samples, failed to observe any differences in alpha and beta diversity, but observed differences in 19 taxa between the participants with low compared to high fatigue. A smaller study by Xiao and colleagues in 13 participants with head and neck cancer who received mixed treatments also reported numerous taxa differences (including those associated with inflammation and lower levels of bacteria that produce SCFA) between those with and without fatigue, although the significance of these was not clearly reported [57]. Finally, Gonzalez-Mercardo and colleagues undertook a study in 50 participants at the end of CRT for colorectal cancer [18] and reported some differences in composition between those with and without fatigue, such as increased *Eubacterium, Streptococcus, Adlercreutzia,* and *Actinomyces*; however, the significance of these was again not clearly reported.

Of the two remaining studies, Deleemans and colleagues investigated fatigue following predominantly CT for mixed cancers and observed no association between fatigue and alpha diversity or composition [52]. Finally, Wei and colleagues compared mild and severe fatigue in 20 participants with advanced lung cancer, and similar to some previous studies, reported no difference in alpha or beta diversity, but various changes in composition between the two groups [58]. Specifically, in patients with mild fatigue, there were lower levels of Enterobacteriaceae family and Escherichia-Shigella genus, both known to lead to increased inflammation, and higher levels of Lachnospiracea, known to increase SCFA production. The significance of other reported differences was again not clearly reported. Taken together, however, these findings, similar to other adverse effects described above, provide the building blocks of evidence associating fatigue severity post-cancer treatment and composition of the gut microbiome.

## 6. Potential Interventions Targeting Gut Microbiome to Modulate Cancer Treatment Adverse Effects

There has been extensive research conducted to determine the efficacy of a variety of treatment options that target the gut microbiome in order to reduce potential adverse effects of cancer therapies, including intense dietary modifications, and pre- and probiotics [45,59,60]. Indeed, the recent guidelines published by the Multinational Association of Supportive Care in Cancer and International Society for Oral Oncology (MASCC/ISOO) [45] discuss a broad range of these options to reduce GI mucositis following cancer treatments. In brief, with regard to the use of probiotics, the most common probiotics for people undergoing cancer treatments that were investigated include *Lactobacillus* and *Bifidobacterium* with regard to the reduction in GI mucositis adverse effects [61,62]. The underlying mechanism of this probiotic benefit is thought to lie not only with the enhancement of GI microflora, but also the creation of a barrier to pathogens by reducing epithelial permeability and lowering intestinal pH, and reducing the number of pathogens through competitive inhibition [61]. In contrast, the evidence regarding the ability of prebiotics and selectively fermented or non-fermented non-digestible foods to reduce GI adverse effects is mixed such that they were reported to either reduce the occurrence of diarrhoea following RT [63] or have no impact on diarrhoea following CRT in people with pelvic cancer [64]. Consequently, the use of probiotics, specifically *Lactobacillus*, is now clinically recommended in GI mucositis treatment guidelines as being beneficial for people with pelvic cancers undergoing RT or CRT; however, there was not sufficient evidence to support guidelines with regard to other dietary modifications [45].

Similar to the GI adverse effects following cancer treatments, there is a limited number of studies that examine the potential impact of interventions on other adverse effects including psychoneurological disorders, fatigue, and cognition. A recent systematic review by Deleemans and colleagues [60] reported on seven studies that investigated the impact of pre- and pro-biotics on quality of life [61,63,64,65,66,67,68], and one study that measured fatigue, anxiety, and depression [67]. In brief, there was no impact of probiotics on quality of life, and whilst prebiotics did not alter quality of life for people undergoing pelvic RT [63,64], they did maintain scores pre- and post-treatment for people head and neck cancer [65]. In contrast, fatigue was lowered in people with colorectal cancer who received probiotics [67]. Whilst most recently, probiotics were reported to decrease CT-induced cognitive changes in people with breast cancer [69].

Another possible intervention that can influence the composition of the gut microbiome in a relatively short period of time is dietary modifications [70]. Consequently, it was suggested that diet should be considered as a potential prevention or treatment for cancer treatment adverse effects [59]. Indeed, a recent review discussed the importance of targeting dietary intake around the production of key bacterial taxa, such as *Bacteriodes* and *Firmicutes,* in order to modulate adverse effects [71]. For example, it is understood that high fibre diets lead to an increased production of SCFA’s [72,73], potentially reducing the prevalence and severity of adverse effects. As previously mentioned, reduced levels of SCFAs lead to compromised epithelial barriers and increased inflammation and oxidative stress [14]. Consequently, dietary changes to enhance SCFA production may substantially impact adverse effects.

In summary, these findings collectively demonstrate the potentially substantial impact that probiotics, and to a lesser extent, prebiotics and other dietary interventions, could have on a multitude of adverse effects of cancer treatments. However, there remains a clinical need for future studies to expand our knowledge regarding the clinical potential across different cancer cohorts and treatment regimens.

## 7. Conclusions

This review highlighted the key evidence suggesting the gut microbiome plays a role in the development of adverse effects following cytotoxic cancer treatments. The extent of the evidence varies substantially, from the well-described role in GI and oral mucositis adverse effects to the building blocks for roles in others, such as psychoneurological, cancer cachexia, and fatigue adverse effects. The latter are still limited by the relatively small number of human clinical studies in a variety of different participant populations with varying degrees of stringency in reporting outcomes. In all areas, the obvious limitations to date include small samples sizes, heterogenous populations, and a lack of replication of the findings in subsequent studies. Consequently, in order to expand our understanding of the likely role of the gut microbiome in the modulation of these adverse effects, further well-designed research with consistency in outcome reporting is encouraged. Furthermore, studies need to incorporate variables known to impact microbial composition, including diet, exercise, region, and ethnicity to make more nuanced judgments and begin to create a knowledge platform to enable intervention for manipulating the microbiome to prevent or better manage these adverse effects. Indeed, gut microbiome engineering as a possible intervention for cancer therapies is a new and exciting area where recent research showed that the efficacy of cancer therapies may be linked to the composition of an individual’s gut microbiome [36]. As such, this emerging area warrants further investigation. For a detailed review on this topic, please see Zhao and colleagues [74]. Nonetheless, in conclusion, this review highlighted that the gut microbiome is a highly dynamic ecosystem associated with numerous adverse effects of cancer treatments. Understanding these associations further may lead to new and effective therapeutic interventions to improve the quality of life of people being treated for cancer.

## Figures and Tables

**Table 1:** Summary of human clinical studies investigating the impact of the gut microbiome on gastrointestinal (GI) and oral mucositis following chemotherapy (CT), radiotherapy (RT), or combination chemoradiotherapy (CRT) for the treatment of various cancers.

GI Mucositis Following CT			
Participants	Study Protocol	Study Outcomes	Reference
Group 1: mixed Ca CT *n* = 16 (6 M, 10 F); HC *n* = 2 (1 M, 1 F). Group 2: mixed Ca CT *n* = 10 (7 M, 3 F); HC *n* = 5 (1 M, 4 F).	Samples: Faecal for all groups. Group 2: Faecal and blood samples; Faecal bacterial growth using qualitative score, DNA extraction and RT-PCR analysis, calprotectin levels; Serum MMP-2, MMP-3, MMP-9, NF-κB, IL-1β and TNF levels; Toxicity scales: NCI common GI tract toxicity criteria.	Bacterial richness, diversity and composition: - no difference; Faecal calprotectin levels: - increased in group 2 CT vs. HC (*p* < 0.05); Serum levels: - increased MMP-3 (*p* < 0.01) 2 and 5 d post-vs pre-treatment; - increased MMP-9 (*p* = 0.02) 2 d post- vs. pre-treatment.	Stringer et al., 2013 [23]
*n* = 28 (18 M, 10 F), non-Hodgkin’s lymphoma	Samples: Faecal: pre- (S1, *n* = 28) and post-CT (S2, *n* = 15); Faecal DNA extraction and 16S rRNA sequencing; Toxicity: GI—abdominal pain, diarrhoea, nausea, vomiting, and scale NR.	Bacterial richness, diversity, and composition: - pre- vs. post-CT phylum: lower abundance Firmicutes (*p* = 0.0002) and Actinobacteria (*p* = 0.002), higher abundance Proteobacteria (*p* = 0.0002), - pre- vs. post-CT genus: lower abundance *Ruminococcus*, *Oscillospira*, *Blautia*, *Lachnospira*, *Roseburia*, *Dorea*, *Coprococcus*, *Anaerostipes*, *Clostridium*, *Collinsella, Adlercreutzia, Bifidobacterium* (*p* < 0.05), higher abundance *Citrobacter, Klebsiella, Enterococcus*, *Megasphaera, and Parabacteroides* (*p* < 0.05), - association between microbiome and GI toxicity NR.	Montassier et al., 2015 [30]
Total *n* = 44 F, breast Ca adjuvant CT *n* = 26; neoadjuvant CT *n* = 18.	Samples: Faecal: pre-CT (T0, *n* = 44), during adriamycin/cyclophosphamide CT (T1, *n* = 43), during docetaxel CT (T2, *n* = 29), one mth post-CT (T3, *n* = 37); Faecal DNA extraction and 16S rRNA analysis; Toxicity scales T0—T3: NCI CTCAE (diarrhoea, peripheral sensory neuropathy, hand-foot syndrome, fatigue, nausea, oral mucositis, vomiting, alopecia, and constipation; toxicity, grade ≥ 1).	Bacterial richness, diversity, and composition: - T0 vs. T3: lower richness (*p* = 0.003), - higher abundance T2 and lower abundance T3 of Proteobacteria (*p* = 0.006), - genus: T3 higher abundance unclassified *Enterobacterales* (*p* < 0.001), *Lactobacillus* (*p* = 0.004), lower abundance *Ruminococcaceae NK4A214 group* (*p* < 0.001), *Marvinbryantia* (*p* = 0.020), *Christensenellaceae R7 group* (*p* = 0.008), and *Ruminococcaceae UCG-005* (*p* < 0.001); Toxicity: - diarrhoea vs. no diarrhoea: lower richness T2 (*p* = 0.04), lower alpha diversity T3 (Shannon index, *p* = 0.006), negative correlation with *Ruminococcaceae UCG-005* (*p* = 0.027), and *Ruminococcaceae NK4A214* (*p* = 0.033), - nausea: negative correlation with richness (*p* = 0.048) and alpha diversity (Shannon index, *p* = 0.029) T3, - no difference in phylum or genus associated with any toxicity at T1 and T2 (*p* ≥ 0.16).	Aarnoutse et al., 2022 [44]
**GI mucositis following RT**			
RT *n* = 10 (2 M, 8 F), mixed Ca - no diarrhoea: *n* = 4 (2 M, 2 F) - diarrhoea: *n* = 6 (6 F) varying grades; HC *n* = 5.	Samples: Faecal: pre-RT (S1), 2nd–3rd wk RT (S2), end RT (S3), 2 wk post-RT (S4); Faecal DNA extraction and 16S rRNA DGGE analysis; Toxicity: diarrhoea CTC score.	Bacterial richness, diversity and composition: - diarrhoea vs. no diarrhoea, HC: modified profile between S3/4 and S1 (*p* < 0.03).	Manichanh et al., 2008 [42]
RT *n* = 11 (2 M, 9 F), pelvic Ca; HC *n* = 4; sex- and age-matched.	Samples: Faecal: pre- and post-RT. Blood: pre- and 3rd wk and 5th wk; Faecal DNA extraction and 16S rRNA Sequencing; Serum citrulline, orosomucoid, haptoglobin, α1-antitrypsin, LPS via ELISA; TNF-α via microfluidic chip; Toxicity: -CTCAE diarrhoea; yes/no occurrence; -MFI-20 fatigue score 0–20.	Bacterial richness, diversity and composition: - diarrhoea vs. no diarrhoea and HC pre-RT: lower alpha diversity (Shannon’s index, *p* < 0.01); higher *Firmicutes/Bacteroidetes* ratio (*p* < 0.05); higher abundance of *Bacteroides*, *Dialister, Veillonella* genus and lower abundance of *Clostridium XI* and *XVIII*, *Faecalibacterium*, *Oscillibacter*, *Parabacteroides*, *Prevotella,* and unclassified genus (*p* < 0.05), - both RT groups post-RT (*p* < 0.05): lower diversity and higher unclassified bacteria; higher abundance Bacteroides and Clostridium_XIVa; lower abundance Faecalibacterium, Lachnospiracea, Oscillibacter, Roseburia, and Streptococcus, - diarrhoea vs. no diarrhoea post-RT (*p* < 0.05): higher *Clostridium XI* and *XVIII* and unclassified; lower *Veilonella*; higher abundance of genera *Alistipes*, *Bacteroides*, *Clostridium_XI*, *Erysipelotrichaceae*, *Escherichia*, *Lachnospiracea*, *Megamonas*, and unclassified; lower abundance of genera *Clostridium_XIVa* and *Sutterella;* Diarrhoea: increased fatigue at wk 3 and 5 RT (*p* < 0.01); Diarrhoea: higher serum TNF-α wk 3 RT (*p* < 0.01), higher LPS wk 5 RT (*p* < 0.05), higher haptoglobin wk 5 RT (*p* < 0.05), and lower citrulline at wk 3 and 5 RT (*p* < 0.01).	Wang et al., 2015 [17]
Total *n* = 18 F, cervical Ca, RT RE *n* = 10; Non-RE *n* = 8.	Samples: Faacal: day prior to and day 1 after RT; Faecal DNA extraction and 16S rRNA sequencing; Toxicity scale: RTOG clinical symptoms abdominal pain, tenesmus, rectal bleeding, faecal incontinence, diarrhoea, vomiting; grade 1, 2, or 3.	Bacterial richness, diversity, and composition RE vs. non-RE: - lower alpha diversity (*p* < 0.006); also decreased alpha diversity severe vs. mild RE (*p* = 0.034), - higher beta diversity (*p* = 0.000), - higher abundance *Proteobacteria* (*p* = 0.03), *Gammaproteobacteria* (*p* = 0.04), *Enterobacteriaceae* (*p* = 0.04), - lower abundance *Bacteroidaceae* (*p* = 0.004), *Ruminococcaceae* (*p* = 0.03), - lower abundance *Bacteroides* (*p* = 0.004), *Blautia* (*p* = 0.01), *Ruminococcaceae_UCG-003* (*p* = 0.048), - pre- vs. post-RT with RE and mild vs. severe RE: higher abundance *Coprococcus* (*p* = 0.034), - low, moderate, and severe RE: differences in *Alcanivorax* (*p* = 0.01), *Coprococcus* (*p* = 0.04), *Collinsella* (*p* = 0.02), *Phenybacterium* (*p* = 0.04), *rc4_4* (*p* = 0.02), and *Virgibacillus* (*p* = 0.008) between low, moderate, and severe RE.	Wang et al., 2019 [24]
Total *n* = 134 M, prostate Ca with RE and without RE Early RE *n* = 32, ≤ 1 y post-RT; Late RE *n* = 87, ≥ 2 y post-RT; Colonoscopy RE: *n* = 9, ≥ 1 y post-RT; Colonoscopy HC: *n* = 6.	Samples: Faecal for all groups. Colonoscopy groups: Intestinal mucosal anterior rectum all plus distal sigmoid from RE participants; Metataxanomics, cytokine analysis, histopathology analysis; Toxicity scales: RTOG: diarrhoea, proctitis; LENT-SOM: sphincter control, tenesmus, bleeding occurrence, pain, and bleeding management; and PRO: bowel subset GI symptom score validated for RE.	Bacterial richness, diversity, and composition: - no association in faecal bacteria with RTOG or LENT-SOM in early or late RE, or with PRO in late RE, - decreased faecal diversity over time associated with PRO in early RE (*p* = 0.03), - no difference between faecal, intestinal diversity in colonoscopy RE vs. HC,- higher faecal *Clostridium IV* with PRO toxicity in early RE (*p* = 0.007), - higher faecal *Roseburia* with RTOG and LENT-SOM toxicity in late RE (*p* < 0.001); Intestinal mucosa cytokine expression, RE vs. HC: - lower: IL-7 (*p* = 0.05), IL-12/IL-23p40 (*p* = 0.03), IL-15 (*p* = 0.05), IL-16 (*p* = 0.009), - higher: eotaxin (*p* = 0.03), - negative correlation: IL-15 and *Roseburia, Propionibacterium* and *Streptococcus* (all *p* < 0.05), - positive correlation: IL-15 and *Parabacterides*, Eotaxin and *Flavonifractor* (both *p* < 0.05).	Reis Ferreira et al., 2019 [25]
**GI mucositis following CRT**			
Total CRT *n* = 22 (6 M, 16 F), rectal Ca no/mild diarrhoea *n*= 14 (4 M, 10 F); severe diarrhoea *n*= 8 (2 M, 6 F).	Samples: Faecal; pre- and post-CRT; Faecal DNA extraction and 16S rRNA sequencing; Toxicity: CTCAE diarrhoea; grade 0/1—no/mild; grade 2+—severe.	Bacterial richness, diversity, and composition severe vs. no/mild diarrhoea: - no differences in richness or diversity, - lower abundance *Butyricicoccus* (*p* = 0.005), *Hungatella* (*p* = 0.025) genus, and 13 species (*p* = 0.011 to 0.049)—*Bacteroides vulgatus*, *Bacteroides xylanisolvens*, *Bacteroides/unclassified* (OTU 00059, OTU 00071, OTU 00077, OTU 00110, OTU 00194), *Blautia/unclassified* (OTU 00190), *Bifidobacterium/unclassified* (OTU 00022), *Flavonifractor plauti*, *Clostridiales/unclassified* (OTU 00114), *Lachnospiraceae/unclassified* (OTU 00192), and *Roseburia/unclassified* (OTU 00038).	Shi et al., 2020 [43]
**Oral mucositis following CT**			
Total *n* = 37 CT, mixed paediatric Ca OM *n* = 25 (19 M, 6 F); non-OM *n* = 12 (9 M, 3 F); HC *n* =37 (28 M and 9F).	Samples: Oral mucosa; pre- and during CT; Oral mucosa DNA extraction and 16S rRNA sequencing; Toxicity: WHO OM; grade 1–4.	Bacterial richness, diversity, and composition: - CT vs. HC: lower diversity (Shannon index, *p* < 0.01) and more heterogenous (*p* < 0.001), - OM vs. non-OM, pre-CT: higher diversity (Shannon index, *p* < 0.05) and more heterogenous (*p* < 0.001); higher *Bacteroidetes* Capnocytophaga (*p* = 0.017), *Firmicutes* (*p* < 0.003), *Fusobacteria* (*p* = 0.027), and *Spirochaetes* (*p* = 0.027) phylum, - OM pre- vs. during CT: increased *Firmicutes Staphylococcus* (*p* < 0.001), decreased *Proteobacteria Derxia* (*p* < 0.027), - non-OM pre- vs. during CT: increased *Proteobacteria Xanthomonas* (*p* = 0.003).	Ye et al., 2013 [11]
**Oral mucositis following RT or CRT**			
Total *n* = 17 (13 M, 4 F), head/neck Ca *n* = 13 RT; *n* = 4 CRT.	Samples: Faecal; pre-treatment; Faecal DNA extraction and 16S rRNA sequencing; Toxicity: - NCI CTCAE OM; grade 1–4.	Bacterial richness, diversity, and composition OM grade 3–4 vs. grade 1–2: - no difference in richness, alpha or beta diversity, - higher *Eubacterium* (*p* = 0.019), *Victivallis* (*p* = 0.016), and *Ruminococcus* (*p* = 0.027) genera, - lower unclassified RF32 genus (*p* = 0.032), - correlation between the relative abundance of *Victivallis* and OM grade (r = 0.67, *p* = 0.003).	Al-Qadami et al., 2023 [16]

Abbreviations: CT—chemotherapy; CRT—chemoradiotherapy; CTC—common terminology criteria; CTCAE—common terminology criteria for adverse events; d—days; DGGE—denaturing gradient gel electrophoresis; F—female; GI—gastrointestinal; HC—healthy controls; IL-1β—interleukin-1 beta; LENT-SOM—Late Effects of Normal Tissues clinician reported outcome; M—male; MFI-20—Modified Multidimensional Fatigue Inventory; MMP—matrix metalloproteinase; mth—months; *n*—number; NCI—National Cancer Institute; NF-κB—nuclear factor kappa B; NR—not reported; OM—oral mucositis; PRO—patient-reported outcomes; RTOG—Radiation Therapy Oncology Group clinician reported outcome; RE—radiation enteritis; rRNA—ribosomal ribonucleic acid; RT—radiotherapy; RT-PCR—real-time polymerase chain reaction; TNF—tumour necrosis factor alpha; vs.—versus; WHO—World Health Organisation; wk—weeks; and y—years.

**Table 2:** Summary of human clinical studies investigating the impact of the gut microbiome on psychoneurological disorders (including fear of cancer recurrence, anxiety, depression, and cognitive impairments) following chemotherapy (CT), radiotherapy (RT), or hormone therapy for the treatment of various cancers.

Participants	Study Protocol	Study Outcomes	Reference
Total *n* = 12 F, breast Ca *n* = 8 CT; *n* = 7 RT.	Samples: Faecal: average 54 (±56) mth post-diagnosis: 0, 3, and 6 mth; Faecal DNA extraction and 16S rRNA sequencing analysis; Psychoneurological scales: Hospital Anxiety and Depression Scale (HADS), Pittsburgh Sleep Quality Index.	Bacterial richness, diversity, and composition: - anxiety and beta diversity: *p* = 0.002, specifically genera *Coprococcus* (*p* = 0.041), *Bacteroides* (*p* = 0.041); Psychoneurological scales: no association between CT and RT and outcomes.	Paulsen et al., 2017 [31] *
Total *n* = 126 (1 M, 125 F), breast Ca *n* = 57 CT; *n* = 69 no CT.	Samples: Faecal: average 65 (±37) mth post-diagnosis; Faecal DNA extraction and 16S rRNA sequencing analysis; Psychoneurological scales: CARS (recurrence); HADS (anxiety, depression, used to adjust bacterial outcomes in statistical analysis).	Bacterial richness, diversity, and composition: - no CT, CARS score and bacterial changes: no association- CT, CARS score and phylum: higher abundance *Bacteroidetes* associated with higher score (*p* = 0.04); higher abundance *Firmicutes* associated with lower score (*p* ≤ 0.03), - CT, CARS score and genus: higher abundance *Bacteroidetes* associated with higher score (*p* < 0.01); higher abundance *Lachnospiraceae.g* (*p* = 0.03), and *Ruminococcus* (*p* = 0.02) associated with lower score, - CT, CARS score and diversity: higher alpha diversity (Shannon’s index) associated with lower score (*p* = 0.04).	Okubo et al., 2020 [19]
Total *n* = 35 *n* = 17 mixed cancer (6 M, 11 F), all CT (18% also RT, 12% also hormone and 6% also immunotherapy); *n* = 18 HC (10 M, 8 F).	Samples: Faecal: *n* = 17 CT (average 16.9 ± 16.4 mth post-CT), *n* = 13 HC; Faecal DNA extraction and 16S rRNA sequencing analysis; Psychoneurological scales: Impact of Life Event Scale (PTSD), NIH PROMIS (depression, anxiety, pain, cognitive function, social isolation, GI outcomes [constipation, diarrhoea, gas, bloating, and abdominal pain]).	Bacterial richness, diversity, and composition: - Cancer < 6 mth vs. Cancer > 6 mth, HC: lower alpha diversity (Chao index, *p* < 0.05), - Cancer vs. HC: higher abundance *Selenomondales* (*p* < 0.05), *Veilloneliaceae* (*p* < 0.05), *Intestinibacter* (*p* = 0.04), lower abundance *Barnesiella* (*p* = 0.03), *Bilophila* (*p* = 0.01), and *Anaerotruncus* (*p* = 0.04), - Cancer: negative correlation between alpha diversity (Chao1 and Shannon Index), and depressive function (*p* < 0.02), positive correlation between alpha diversity (Chao1 and Shannon Index) and cognitive function (*p* < 0.05), - Cancer correlation between bacterial taxa and outcomes: Negative—*Lachnospiraceae* (ASV_4) and anxiety (*p* = 0.02), and PTSD symptoms (*p* = 0.05), (ASV_15) and cognitive function (*p* = 0.04); *Ruminococcaceae* (ASV_10) and depressive functional (*p* = <0.001), and social isolation (*p* = 0.01); *Intestinibacter* (ASV_41), and depressive functional (*p* = 0.05). Positive—*Lachnospiraceae* (ASV_26) and diarrhoea (*p* = 0.03); *Intestinibacter* (ASV_41) and cognitive function (*p* = 0.02); Cancer vs. HC: higher GI symptoms (*p* < 0.05), anxiety, depression (*p* < 0.01), PTSD (*p* < 0.01), pain (*p* < 0.05), social isolation (*p* < 0.01); and lower cognitive function (*p* < 0.01).	Deleemans et al., 2022 [52] *
Total *n* = 35 F, breast Ca CT *n* = 21; HC *n* = 14.	Samples: Faecal: *n* = 17 CT (average 13 days post-CT), *n* = 13 HC; Faecal DNA extraction and 16S rRNA sequencing analysis; Psychoneurological scales FACT-Cog (cognition), CES-D (depression), PROMIS (mental health), and PSS (stress perception).	CT vs. HC: lower FACT-Cog scores = more cognitive issues (*p* < 0.001), higher CES-D scores = more depression (*p* = 0.03); Bacterial richness, diversity, and composition: - CT vs. HC: lower abundance Verrucomicrobia phylum (*p* = 0.02), genus *Akkermansia* (*p* = 0.02), higher abundance *Clostridium, Actinobacillus* genus (*p* = NR), - FACT-Cog score high vs. low: higher abundance of *Odoribacter* genus, *Clostridium* genus, and Erysipelotrichaceae family (*p* = NR), - CES-D correlated with Tenericutes phylum (*p* = 0.002).	Bilenduke et al., 2022 [20]
Total *n* = 70 F, breast Ca no obesity *n* = 38 (BMI ≤ 29.9), *n* = 27 CT, *n* = 22 RT, and *n* = 15 hormone therapy * unknown if combined therapy; obesity *n* = 32 (BMI ≥ 30.0), *n* = 18 CT, *n* = 22 RT, and *n* = 13 hormone therapy * unknown if combined therapy.	Samples: Faecal: no obesity average 6.3 ± 6.0 y post-diagnosis; obesity average 4.4 ± 3.0 y post-diagnosis; Faecal DNA extraction and 16S rRNA sequencing analysis; Psychoneurological scales: health-related quality of life SF-36 score (physical and mental health).	Bacterial richness, diversity, and composition, no obesity vs. obesity: - higher abundance *Ruminococcus* (*p* = 0.003), *Streptococcus* (*p* = 0.049), *Roseburia* (*p* = 0.035), and *Dorea* (*p* = 0.003), *Fusobacterium* (*p* = 0.019), Enterobacteriaceae (*p* = 0.035), - lower abundance *Pseudomonas* (*p* = 0.016), *Proteus* (*p* = 0.017), and *Sutterella* (*p* = 0.02), - negative correlations between psychoneurological scales and bacterial composition after BMI adjustment: *Ruminococcus* and physical functioning (*p* = 0.036), vitality (*p* = 0.012), mental health (*p* < 0.001); *Dorea* and mental health (*p* = 0.006), social functioning (*p* = 0.009), and mental component summary score (*p* = 0.006).	Smith et al., 2023 [53]

Abbreviations: BMI—body mass index; Ca—cancer; CARS—Concerns About Recurrence Scale; CES-D—Center for Epidemiological Studies-Depression; CT—chemotherapy; F—female; FACT-Cog—Functional Assessment of Cancer Therapy-Cognitive function; GI—gastrointestinal; HADS—Hospital Anxiety and Depression Scale; HC—healthy controls; M—male; mth—months; *n*—number; NCI—National Cancer Institute; NIH—National Institutes of Health; NR—not reported; PROMIS—Patient-Reported Outcomes Measurement Information System; PSS—Perceived Stress Scale; rRNA—ribosomal ribonucleic acid; RT—radiotherapy; SF-36—Short-form Health Survey self-report; vs.—versus; and y—years. * Indicates study is also listed in Table 4.

**Table 3:** Summary of human clinical studies investigating the impact of the gut microbiome on cancer cachexia prior to (baseline) or following treatment of various cancers.

Participants	Study Protocol	Study Outcomes	Reference
Total *n* = 31 (19 M, 12 F), lung Ca C, malnourished *n* = 8 + severely malnourished *n* = 4 (5 M, 7 F); NC, *n* = 19 (14 M, 5 F).	Samples baseline: Faecal; Blood; Faecal DNA extraction and sequencing; plasma metabolomic; C score: aPG-SGA.	Bacterial richness, diversity and composition C vs. NC: - no difference in alpha diversity, - no difference in *Firmicutes/bacteroidetes* ratio, - 44 species differences: including lower abundance *Prevotella copri* (FDR-corrected *p* = 0.006); C vs. NC: lower survival probability (*p* = 0.005).	Ni et al., 2021 [55]
Total *n* = 183 (80 M, 103 F) C *n* = 33 (13 M, 20 F), mixed Ca; NC *n* = 74 (13 M, 61 F), mixed Ca; HC *n* = 76 (54 M, 22 F).	Samples baseline: Faecal; Faecal DNA extraction and sequencing, SCFA and BCFA analysis, calprotecin levels; C classification: > 5% body weight loss past 6 mth.	Bacterial richness, diversity and composition C vs. NC, HC: - no difference in alpha or beta diversity (*p* > 0.053), - Phylum: higher abundance Proteobacteria phylum (*p* < 0.001), - Genus: lower abundance of *Megamonas* (*p* < 0.05), *Peptococcus* (*p* < 0.001); higher abundance of *Enterobacteriaceae* (unknown, *p* < 0.01), *Veillonella* (*p* < 0.001); negative association between *Enterobacteriaceae* (unknown) and *Veillonella* co-occurrence, vs. positive in NC or no association in HC; Faecal metabolomics C vs. NC: - lower acetate (*p* < 0.05), negative correlation with *Peptococcus* and *Enterobacteriaceae* (unknown) abundance (both *p* < 0.01), - no difference in other SCFA or BCFA, calprotecin.	Ubachs et al., 2021 [21]
Total *n* = 113 (72 M, 41 F), lung Ca C *n* = 57 (34 M, 23 F) - ICI *n* = 42 (74%) - ICI + CT *n* = 15 (26%) NC *n* = 56 (38 M, 18 F) - ICI *n* = 31 (55%) - ICI + CT *n* = 25 (45%) *p* < 0.05 treatments vs. C	Samples baseline (between 1 wk prior to and after ICI start): Faecal; Faecal DNA extraction and 16S rRNA sequencing; C classification: baseline: > 5% weight loss or BMI < 20 + > 2% weight loss past 6 mth, - reversible during ICI: > 5% or if BMI < 20, >2% weight gain between baseline and ICI, - irreversible during ICI: all others; NC baseline: - latent during ICI: > 5% weight loss or if BMI < 20, >2% weight loss during ICI, - free during ICI: all othersPFS, OS: RECIST guidelines.	Bacterial richness, diversity and composition C vs. NC baseline: - no difference in alpha diversity, - different beta diversity (*p* = 0.003), - taxa from LDA scores: 16 higher abundance, including *Escherichia-Shigella*, *Christensenellaceae R-7*, *Cellulosilyticum*, *Hungatella*; 14 lower abundance, including *Anaerostipes*, *Agathobacter, Blautia, Dorea Eubacterium halli*, and *Eubacterium ventriosum* (*p* = NR); Clinical outcomes C vs. NC: - C vs. NC baseline: lower PFS (*p* = 0.003), OS (*p* = 0.02), - C baseline and reversible, vs. irreversible C during ICI: longer PFS (*p* = 0.0042), OS (*p* = 0.027), - NC baseline and latent, vs. free C during ICI: no difference in PFS or OS.	Hakozaki et al., 2022 [22]

Abbreviations: aPG-SGA—abridged patient-generated subjective assessment score; BCFA—branched-chain fatty acids; BMI—body mass index; C—cachectic; Ca—cancer; CT—chemotherapy; F—female; HC—healthy control; ICI—immune checkpoint inhibitors; LDA—linear discriminant analysis; M—male; mth—months; NC—non-cachectic; NR—not reported; OS—overall survival; PFS—progression-free survival; RECIST—response evaluation criteria in solid tumours; SCFA—short-chain fatty acids; vs.—versus; and wk—weeks.

**Table 4:** Summary of human clinical studies investigating the impact of the gut microbiome on fatigue following chemotherapy (CT), radiotherapy (RT), or combination chemoradiotherapy (CRT) for the treatment of various cancers.

Participants	Study Protocol	Study Outcomes	Reference
Total *n* = 12 F, breast Ca *n* = 8 CT; *n* = 7 RT.	Samples: Faecal: average 54 (±56) mth post-diagnosis: 0, 3, and 6 mth; Faecal DNA extraction and 16S rRNA sequencing analysis; Fatigue score: Fatigue Symptom Inventory.	Bacterial richness, diversity, and composition: - fatigue and diversity: higher alpha (Shannon’s index *p* = 0.005); beta diversity (*p* = 0.01), specifically genera *Faecalibacterium* (*p* = 0.033), *Prevotella* (*p* = 0.044).	Paulsen et al., 2017 [31] *
Total *n* = 88 (43 M, 45 F), mixed Ca Low fatigue *n* = 58 (25 M, 33 F); High fatigue *n* = 30 (18 M, 12 F).	Samples: Faecal: baseline; Faecal DNA extraction and 16S rRNA sequencing analysis; Fatigue score: MDASI-immunotherapy baseline, low = 0–4, high = 5–10.	Bacterial richness, diversity and composition high vs. low fatigue: - no difference in alpha and beta diversity, - 19 taxa different, including lower abundance *Eubacterium Hallii* (*p* = 0.033), higher abundance *Cosenzaea* (*p* = 0.004).	Hajjar et al., 2021 [56]
Total *n* = 13 (11 M, 2 F), head/neck Ca Low fatigue *n* = 6 (5 M, 1 F); High fatigue *n* = 7 (6 M, 1 F); Prior exposure to mixed therapies, CT, and RT.	Samples: Faecal: baseline and 1 mth post-RT; Blood; Faecal DNA extraction and 16S rRNA sequencing analysis; Fatigue score: MFI-20 baseline and 1 mth post-RT.	Bacterial richness, diversity and composition high vs. low fatigue: - lower abundance: Firmicutes, family *Ruminococcaceae*, genus *Subdoligranulum* and Faecalibacterium, species *uncultured Firmicutes bacterium;* Firmicutes, genus *Agathobacter*, *Lactococcus*, species *Ruminococcus* sp. *N15.MGS-57;* genus *Bifidobacterium,* species *Desulfovibrio fairfieldensis* (*p* = NR).	Xiao et al., 2021 [57]
Total *n* = 50 (28 M, 22 F), rectal Ca Fatigue *n* = 35 (20 M, 15 F); No fatigue *n* = 15 (8 M, 7 F).	Samples: Faecal: end CRT; Faecal DNA extraction and 16S rRNA sequencing analysis; Fatigue score: PROMIS-F end CRT.	Bacterial richness, diversity and composition fatigue vs. no fatigue: - higher abundance of *Eubacterium, Streptococcus* (family *Streptococcaceae*), *Adlercreutzia*, and *Actinomyces* genus (family *Actinomycetaceae*) (*p* = NR).	González-Mercado et al., 2021 [18]
Total *n* = 35 *n* = 17 mixed Ca (6 M, 11 F), all CT (18% also RT, 12% also hormone, and 6% also immunotherapy); *n* = 18 HC (10 M, 8 F).	Samples: Faecal: *n* = 17 CT (average 16.9 ± 16.4 mth post-CT), *n* = 13 HC; Faecal DNA extraction and 16S rRNA sequencing analysis; Fatigue score: NIH PROMIS fatigue.	Bacterial richness, diversity, and composition, fatigue score: - Ca: no correlation with alpha diversity (Chao1, Shannon index) or composition, - HC: negative correlation with *Ruminococcaceae* (ASV_2) (*p* = 0.01); positive correlation with *Lachnospiraceae* (ASV_54) (*p* = 0.01); Cancer vs. HC: higher fatigue (*p* < 0.01).	Deleemans et al., 2022 [52] *
Total *n* = 20 (15 M, 5 F), lung Ca, CT Mild fatigue *n* = 10 (6 M, 4 F); Severe fatigue *n* = 10 (9 M, 1 F).	Samples Faecal: baseline; Faecal DNA extraction and 16S rRNA sequencing analysis; Fatigue score: Piper Fatigue Scale baseline, mild = 1–3, moderate = 4–6, and severe = 7–10.	Bacterial richness, diversity and composition mild vs. severe fatigue: - no difference in alpha or beta diversity, phyla, - LDA scores: lower abundance class Bacilli (*p* = NR), order Lactobacillales (*p* = NR), order Enterobacteriales (*p* = NR), family *Enterobacteriaceae*, genus *Escherichia-Shigella* (*p* < 0.05), genus *Cetobacterium* (*p* = NR); higher abundance genus *Lachnospiraceae-UCG-008* and family *Lachnospiraceae* (*p* < 0.05).	Wei et al., 2023 [58]

Abbreviations: Ca—cancer; CRT—chemoradiotherapy; CT—chemotherapy; F—female; LDA—linear discriminant analysis; M—male; MDASI—MD Anderson Symptom Inventory-immunotherapy module; MFI-20—Multidimensional Fatigue Inventory; mth—months; *n*—number; NIH—National Institutes of Health; NR—not reported; PROMIS/PROMIS-F—Patient-Reported Outcome Measures Information System fatigue; rRNA—ribosomal ribonucleic acid; RT—radiotherapy; and vs.—versus. * Indicates study is also listed in Table 2.

## Supplementary Materials

The following supporting information can be downloaded at: https://www.mdpi.com/article/10.3390/cancers15174301/s1. Table S1: Key search terms used during the semi-structured literature search for relevant papers in the PubMed, Embase, Emcare, and Cochrane databases.
