# Supplementary material for: The Association between the Gut Microbiome and Development and Progression of Cancer Treatment Adverse Effects
Source: Cancers (Basel). 2023 Aug 28;15(17):4301. doi: 10.3390/cancers15174301 (PMC10487104; doi:10.3390/cancers15174301)
Supplement: Supplementary file 1 [file cancers-15-04301-s001.zip › cancers-2499507-supplementary.pdf]

Supplementary Table S1: Key search terms used during the semi-structured literature search for relevant papers in the PubMed, Embase, Emcare, and Cochrane databases.

**Participants**	**Studies**	**Gut microbiome**	**Adverse effects**	**Cancer treatments**
"neoplasms" [mh] OR Cancer*[tw] OR leukaemia[tw] OR leukemia[tw] OR oncolog*[tw] OR tumor*[tw] OR Tumour*[tw] OR carcinoma*[tw] OR non-squamous cell carcinoma [tw] OR NSCC[tw] OR neoplasm*[tw]	Clinical trial[pt] OR interven*[tw] OR trial [tw] OR “randomised control trial” [tw] OR “pilot stud*” [tw] OR “cohort stud*” [tw]	"Gastrointestinal Microbiome"[mh] OR gut microbio*[tw] OR gut microbiome*[tw] OR gut flora[tw] OR gastric microbio*[tw] OR intestinal flora[tw] OR intestinal microbiota*[tw] OR enteric bacteria[tw] OR gastrointestinal microbio*[tw]	"mucositis"[mh] OR "fatigue"[mh:noexp] OR "Diarrhea"[mh:noexp] OR "cognitive dysfunction"[mh] OR "anxiety"[mh] OR "depression"[mh] OR "mental health"[mh] OR “cancer cachexia” [tw] OR fatigue[tw] OR mucositis[tw] OR GIT mucositis[tw] OR gastrointestinal mucositis[tw] OR oral mucositis[tw] OR diarrhoea[tw] OR diarrhea[tw] OR psychoneurological[tw] OR cognit*[tw] OR "fear of recurrence"[tw] OR anxiet*[tw] OR depres*[tw] OR mental health[tw]	"Induction Chemotherapy"[mh] OR "Induction Chemotherapy/adverse effects"[mh] OR "Chemotherapy, Adjuvant"[mh] OR "Chemotherapy, Adjuvant/adverse effects"[mh] OR "maintenance chemotherapy"[mh] OR "maintenance chemotherapy/adverse effects"[mh] OR "radiotherapy"[mh] OR "immunotherapy"[mh] OR chemotherap*[tw] OR radiotherap*[tw] OR immunotherap*[tw]
